# PTEN inhibitor VO-OHpic attenuates GC-associated endothelial progenitor cell dysfunction and osteonecrosis of the femoral head via activating Nrf2 signaling and inhibiting mitochondrial apoptosis pathway

**DOI:** 10.1186/s13287-020-01658-y

**Published:** 2020-03-30

**Authors:** Xudong Yao, Shengnan Yu, Xingzhi Jing, Jiachao Guo, Kai Sun, Fengjing Guo, Yaping Ye

**Affiliations:** 1 Department of Orthopedics, Tongji Hospital, Tongji Medical College, Huazhong University of Science and Technology, Wuhan, 430030 Hubei China; 2 Department of Oncology, Tongji Hospital, Tongji Medical College, Huazhong University of Science and Technology, Wuhan, Hubei China; 3 Department of Orthopedics, Shandong Provincial Hospital, Shandong University, Jinan, Shandong China

**Keywords:** Glucocorticoid-associated osteonecrosis of the femoral head, Endothelial progenitor cells, Angiogenesis, Apoptosis, Nrf2

## Abstract

**Background:**

Glucocorticoid (GC)-associated osteonecrosis of the femoral head (ONFH) is the most common in non-traumatic ONFH. Despite a strong relationship between GC and ONFH, the detailed mechanisms have remained elusive. Recent studies have shown that GC could directly injure the blood vessels and reduce blood supply in the femoral head. Endothelial progenitor cells (EPCs), which were inhibited quantitatively and functionally during ONFH, play an important role in maintaining the normal structure and function of vascular endothelium. Phosphatase and tensin homolog (PTEN) is a tumor suppressor gene that promotes cell apoptosis, and its expression was found to be elevated in GC-associated ONFH patients. However, whether direct inhibition of PTEN attenuates GC-associated apoptosis and dysfunction of the EPCs remains largely unknown.

**Methods:**

We investigated the effect of, VO-OHpic, a potent inhibitor of PTEN, in attenuating GC-associated apoptosis and dysfunction of EPCs and the molecular mechanism. SD rats were used to study the effect of VO-OHpic on angiogenesis and osteonecrosis in vivo.

**Results:**

The results revealed that methylprednisolone (MPS) obviously inhibit angiogenesis of EPCs by inducing apoptosis, destroying the normal mitochondrial structure, and disrupting function of mitochondria. VO-OHpic treatment is able to reverse the harmful effects by inhibiting the mitochondrial apoptosis pathway and activating the NF-E2-related factor 2 (Nrf2) signaling. Si-Nrf2 transfection significantly reduced the protective effects of VO-OHpic on EPCs. Our in vivo studies also showed that intraperitoneal injection of VO-OHpic obviously attenuates the osteonecrosis of the femoral head induced by MPS and potently increases the blood supply in the femoral head.

**Conclusion:**

Taken together, the data suggests that inhibition of PTEN with VO-OHpic attenuates apoptosis and promotes angiogenesis of EPCs in vitro via activating Nrf2 signaling pathway and inhibiting the mitochondrial apoptosis pathway. Moreover, VO-OHpic also mitigates GC-associated ONFH and potentiates angiogenesis in the femoral head.

## Background

Glucocorticoid (GC) has been widely used in the treatment of many rheumatic and autoimmune diseases. However, high-dose or long-term use of GC could lead to GC-associated osteonecrosis of the femoral head (ONFH) [1]. It is estimated that non-traumatic ONFH cases were 8.12 million among Chinese people aged 15 years and over [2]. Among patients who were diagnosed with non-traumatic ONFH, 26.35% of males and 55.75% of females reported a history of GC use [2]. Researchers proposed several mechanisms for the development of ONFH. Among those hypotheses, vascular hypothesis seems to be the most compelling one. This hypothesis presumes that damaged vessels and decreased local blood flow in the femoral head plays a pivotal role in the pathogenesis of ONFH [3, 4]. GC could decrease the number of blood vessels, reduce blood supply in the femoral head, and cause osteonecrosis [5]. Endothelial progenitor cells (EPCs), as the precursor cells of endothelial cells, play an important role in maintaining the normal structure and function of vascular endothelium. Several studies demonstrated that in patients with GC-associated ONFH, the number and function of EPCs in the blood are impaired [6, 7].

Phosphatase and tensin homolog (PTEN) is a crucial regulator that controls many physiological processes including cell proliferation, differentiation, and apoptosis [8, 9]. PTEN could dephosphorylate proteins and phosphoinositides which are generated by phosphatidylinositol 3-kinase (PI3K), thus counteracting the PI3K/AKT signaling pathway to promote cell apoptosis [10]. Deregulation of the PTEN/PI3K/AKT pathway has been shown to be associated with diseases such as cancers, neurodegenerative diseases, and adverse cardiovascular events [11–13]. PTEN expression was significantly increased in the ischemia/reperfusion myocardial tissue, and PTEN overexpression enhanced Bax expression and promoted apoptosis of the rat myocardial cells [14]. PTEN overexpression could significantly abolish astragaloside IV-induced angiogenesis after myocardial infarction [15]. VO-OHpic is a vanadium-based reversible and noncompetitive inhibitor of PTEN [16]. Inhibition of PTEN with VO-OHpic could attenuate apoptosis and cardiac remodeling in doxorubicin-induced cardiomyopathy [17]. Interestingly, a recent study identified that PTEN was significantly elevated in GC-associated ONFH patients and was identified as a candidate serum biomarker for these patients [18]. We are especially interested in whether direct inhibition of PTEN with VO-OHpic could protect the EPCs from GC-induced damage and further whether VO-OHpic could protect the blood supply in the femoral head of GC-associated ONFH in rats.

Nuclear factor erythroid 2-related factor 2 (Nrf2) controls the expression of many antioxidant enzymes [19]. It could translocate from the cytoplasm to nucleus and then bind with the antioxidant response element (ARE) of targeted antioxidant enzyme genes and lead to their activation [20, 21]. Those downstream antioxidant enzymes include heme oxygenase (HO-1), NADPH quinone dehydrogenase 1 (NQO1), and thioredoxin (Trx) [22, 23]. Nrf2 signaling pathway is activated when cells were under stress and plays a critical role in maintaining cellular homeostasis [24]. Recent studies have shown that PI3K/AKT pathway participates in the activation of Nrf2 [25]. Consistently, PTEN could significantly repress the activity of Nrf2 [26] while loss of PTEN could lead to the activation of Nrf2 [27].

Reactive oxygen species (ROS) are byproducts of normal mitochondrial metabolism [28]. In physiological condition, low level of ROS participates in essential cellular signaling pathways. However, under environmental stress, ROS generation will be excessive and cause oxidative stress, leading to damage of intracellular molecules, disruption of cellular homeostasis, and cellular dysfunction [29, 30]. Many investigations have also confirmed that there is a direct relationship between the mitochondrial apoptotic pathway and mitochondrial ROS [31]. Since Nrf2 signaling promotes the activation of many antioxidant proteins upon oxidizing conditions, it could also protect cells against the effects of mitochondrial ROS [32].

In the present study, we aimed to investigate three problems: (1) whether GC could induce mitochondrial-mediated apoptosis and inhibit angiogenesis of EPCs, (2) whether inhibition of PTEN with VO-OHpic could ameliorate the detrimental effects of GC via activating Nrf2 signaling pathway and inhibiting the mitochondrial apoptosis pathway, and (3) whether intraperitoneal injection of VO-OHpic could promote angiogenesis and inhibit osteonecrosis in GC associated ONFH in rats.

## Methods

### Reagents

MPS was purchased from Pfizer and dissolved in sterile normal saline. VO-OHpic was purchased from selleck (USA, S8174) and dissolved in DMSO. And the final concentration of DMSO is the same (0.1%) in each group including the control. CD31 antibody (UK, ab182981), CD34 antibody (UK, ab81289), VEGF Receptor 2 (VEGFR2) antibody (UK, ab11939), VEGF Receptor 1 (VEGFR1) antibody (UK, ab32152), CD133 antibody (UK, ab19898), SIRT-1 antibody (UK, ab189494), NQO-1 antibody (UK, ab80588), and Trx antibody (UK, ab133524) were purchased from Abcam. FITC-conjugated mouse anti-rabbit IgG antibody was purchased from Boster (China, BM2012). Bad antibody (USA, #9268, for IP), Phospho-Bad antibody (USA, #5284), Bcl-xL antibody (USA, #2764), AKT antibody (USA, #4691), p-AKT antibody (USA, #4060), cleaved caspase-3 (Asp175) antibody (USA, #9661), cleaved caspase-9 antibody (USA, #9508), MFF antibody (USA, #84580), and normal rabbit IgG (USA, #2729) were purchased from Cell Signaling Technology. Bax antibody (USA, 50599-2-Ig, for western blot), Cytochrome C antibody (USA, 66264-1-Ig), Nrf2 antibody (USA, 16396-1-AP), Bcl-xL antibody (USA, 26967-1-AP), Bad antibody (USA, 10435-1-AP), FIS1 antibody (USA, 10956-1-AP), VEGF antibody (USA, 19003-1-AP), HO-1 antibody (USA, 10701-1-AP), and GAPDH antibody (USA, 60004-1-Ig) were purchased from Proteintech.

### Culture and characterization of endothelial progenitor cells (EPCs)

Rat EPCs were isolated and cultured as described before [33]. In brief, rat heart vasculature was removed and perfused with Krebs-Ringer buffer containing 0.06% collagenase. Cells were then collected from the recirculation medium and cultured in M199 culture medium supplemented with 10% fetal bovine serum (FBS), 1% endothelial cell growth supplements, penicillin, and streptomycin. Until 80% confluence, cells were detached and sorted by DAKO Cytomation MoFlo High-Speed Cell Sorter (DAKO, Denmark). Then a single cell was seeded in a 96-well plate. One week later, wells with more cells were selected and cultured further. After five passages, the phenotype of EPCs was identified using flow cytometry analysis. EPCs were washed with PBS three times, then cells were digested and resuspended at a concentration of 1 × 10^6^ cells/ml. EPCs were incubated at room temperature for 1 h with the following antibodies: anti-CD34 antibody, anti-VEGF Receptor 2 (VEGFR2) antibody, and anti-CD133 antibody. EPCs were then incubated with FITC-conjugated mouse anti-rabbit IgG antibody in the dark at room temperature for 30 min, washed with PBS for three times, and resuspended in 300-μl PBS, and cell fluorescence intensity was determined with FACS Calibur flow cytometer (BD, Franklin Lakes, USA). EPCs that were incubated with normal rabbit IgG were selected as negative controls.

### Cell treatment and siRNA transfection

EPCs were treated with MPS combined with or without VO-OHpic for 48 h. SiRNA targeting Nrf2 mRNA were synthesized and purchased from RiboBio Co., Ltd., China. The targeted sequences of siNrf2 were as follows: GGATGAAGAGACCGGAGAA (si-Nrf2-1), CAAACAGAATGGACCTAAA (si-Nrf2-2), and GCAAGAAGCCAGATACAAA (si-Nrf2-3). In brief, 100-nM siRNA were transfected into EPCs by lipofectamine 3000 (Invitrogen, USA, L3000015). After incubation for 24 h, the culture medium was replaced and EPCs transfected with siNrf2 or siControl were treated with 50-μM MPS combined with 1-μM VO-OHpic. The efficiency of siRNA transfection was also measured with endogenous Nrf2 mRNA expression, and western blot analysis was performed to investigate the protein expression after transfection.

### Cell viability assay

The viability of EPCs was determined with a cell counting kit-8 (Boster, China, AR1160). EPCs were seeded in 96-well plates at a density of 3 × 10^3^ cells/well and treated with different concentrations of MPS (0, 10, 20, 50 μM) for 48 h or treated with 50-μM MPS added with different concentrations of VO-OHpic (0, 0.01, 0.1, 1, 10 μM) for 48 h. Finally, 10 μl of CCK-8 solution was added to each well at 37 °C for 4 h to form MTT formazan. A microplate reader (Thermo Fisher Scientific, Vantaa, Finland) was used to measure the absorbance at 450 nm. The optical density (OD) values were used to evaluate the cell viability.

### EPC apoptosis evaluated by Annexin V-FITC/PI staining

EPCs were seeded in 6-well plates at a density of 3 × 10^5^ cells per well. After 24 h, EPCs were treated with 50-μM MPS or 50-μM MPS combined with 1-μM VO-OHpic or left untreated (served as control group). After different treatments, EPCs were washed three times with PBS and harvested to centrifuge tubes. Experiment was then performed using Annexin V-FITC/PI Apoptosis Detection Kit (Beyotime, China, C1062) according to manufacturer’s protocol. In brief, cells were stained with Annexin V-FITC/PI for 15 min at room temperature in the dark. PI−/Annexin V+ and PI+/Annexin V+ EPCs were considered as early and late phase of apoptotic cells, respectively. The results were analyzed with FACS Calibur flow cytometer (BD, Franklin Lakes, USA).

### Immunoprecipitation for Bcl-xL/Bad-binding activity

In brief, EPCs were washed three times with PBS and total proteins were harvested by the NP-40 immunoprecipitation (IP) cell lysis buffer (Boster, China, AR0107). The protein complex was collected with PureProteome protein G magnetic beads (Millipore, MA, LSKMAGG10) according to the manufacturer’s protocol. Proteins were incubated with anti-Bad antibody, anti-Bcl-xL antibody, or control normal rabbit IgG at 4 °C overnight with a dilution ratio of 1:50 respectively. After washed for three times, the beads were added to SDS-loading buffer and boiled for 5 min. The samples were then loaded to SDS-PAGE gel, and the protein level of Bcl-xL and Bad was detected by western blot as described below.

### Mitochondrial specific fluorescence staining

EPCs were initially seeded in 6-well plates at a density of 3 × 10^5^ cells/well. After 24 h, EPCs were treated with 50-μM MPS or 50-μM MPS combined with 1-μM VO-OHpic for 48 h. After treatment, EPCs were washed with PBS and stained with 20-nM Mito-Tracker Green (Beyotime, China, C1048) working solution for 30 min at 37 °C in the dark according to the manufacturer’s protocol. Shapes of mitochondria in EPCs were observed under the fluorescence microscope (EVOS FL Auto, Life Technologies, USA). For the immunofluorescence staining experiment, MitoTracker Red CMXRos (Invitrogen, USA, M7512) was used to stain the mitochondria because of its ability to maintain fluorescence after fixation. After fixation with 4% paraformaldehyde, EPCs were stained with MitoTracker Red following the manufacturer’s protocols. Then EPCs were subjected to further immunofluorescence staining.

### Immunofluorescence staining

After staining with the MitoTracker Red, EPCs were washed three times with PBS and fixed with 4% paraformaldehyde for 20 min at room temperature. After permeabilized with 0.2% triton X-100 for 20 min, cells were blocked for 1 h with 5% BSA at room temperature. EPCs were then incubated with a mixture of rabbit anti-Bax antibody and mouse anti-Cytochrome C antibody overnight at 4 °C. After washing three times with PBS, EPCs were incubated with a mixture of Alexa Fluor 350-labeled rabbit secondary antibody or FITC-conjugated mouse secondary antibody at room temperature for 1 h in the dark. For Nrf2 staining, EPCs were incubated with anti-Nrf2 antibody overnight at 4 °C and further incubated with Cy3-conjugated rabbit secondary antibody for 1 h at room temperature in the dark. Finally, images were captured under a fluorescence microscope.

### Transmission electron microscopy (TEM)

EPCs were covered with 1 ml of 2.5% glutaraldehyde and harvested to centrifuge tubes by cell scrapers. After centrifugation at 300 g for 10 min, cell clumps were then fixed with 2.5% glutaraldehyde for 2 h at room temperature and post-fixed in osmium tetroxide. After embedded in Epon epoxy resin, cell clumps were cut into 60–80-nm sections. The ultrathin sections were stained with lead citrate and uranyl acetate and observed using a transmission electron microscope (HT7700-SS, Tokyo, Japan).

### Detection of intracellular reactive oxygen species (ROS)

EPCs were first seeded in 6-well plates at a density of 3 × 10^5^ cells/well. After 24 h, cells were treated by 50-μM MPS or 50-μM MPS combined with 1-μM VO-OHpic for 48 h. EPC- or si-Nrf2-transfected EPCs were also treated with 50-μM MPS combined with 1-μM VO-OHpic for 48 h. After treatment in different groups, the intracellular ROS level was measured with the Reactive Oxygen Species Assay Kit (Beyotime, China, S0033) according to manufacturer’s instructions. In brief, EPCs were washed with PBS three times and treated with 10-μM DCFH-DA for 20 min at 37 °C in the dark. After incubation, EPCs were washed with PBS and observed under the fluorescence microscope (EVOS FL Auto, Life Technologies, USA). To quantify ROS levels, EPCs were also collected, resuspended, and treated with 10-μM DCFH-DA for 20 min at 37 °C in the dark. After incubation, cells were rinsed with serum-free DMEM/F12, and finally, the mean fluorescence intensity (MFI) was measured with FACS Calibur flow cytometer (BD, Franklin Lakes, USA).

### Detection of mitochondrial membrane potential

EPCs were initially seeded in 6-well plates at a density of 3 × 10^5^ cells/well. After 24 h, EPCs were treated with 50-μM MPS or 50-μM MPS combined with 1-μM VO-OHpic for 48 h. After treatment, the mitochondrial membrane potential was detected with mitochondrial membrane potential assay kit with JC-1 (Beyotime, China, C2006) according to manufacturer’s instruction. In brief, EPCs were washed with PBS and stained with JC-1 staining solution for 20 min at room temperature in the dark. After washing with JC-1 buffer, EPCs were observed under the fluorescence microscope (EVOS FL Auto, Life Technologies, USA). JC-1 exhibits a potential-dependent accumulation in mitochondria, and mitochondrial with normal membrane potential was accumulated with high concentration of JC-1 aggravates and produce red fluorescence. Under circumstances of cell apoptosis, the mitochondrial membrane potential collapsed and JC-1 failed to aggregate in the mitochondria. JC-1 then existed in the form of monomer, and the mitochondria were labeled in green. After treatment, cells were also resuspended with culture medium and the green fluorescence intensity was measured with FACS Calibur flow cytometer (BD, Franklin Lakes, USA).

### Nuclear and cytoplasmic protein isolation

Nuclear and cytoplasmic protein extraction kit (Beyotime, China, P0028) were used to separately extract the nuclear and cytoplasmic protein according to manufacturer’s protocol. The nuclear and cytoplasmic proteins were then respectively subjected to western blot analysis. Lamin B was used as an internal reference for the nuclear protein, and GAPDH was used as an internal control for the cytoplasmic protein.

### Matrigel tube formation assay

After thawed on ice, 100 μl of Matrigel (BD Biosciences, USA) was applied to each well and re-solidified at 37 °C for 30 min. EPCs were pre-treated with 50-μM MPS or 50-μM MPS combined with 1-μM VO-OHpic for 48 h. Then 4 × 10^4^ EPCs were seeded to each well and incubated at 37 °C. After 12 h, the cytoskeleton of EPCs was stained with FITC-conjugated phalloidin (Beyotime, China, C1033). The cell morphology was observed and captured using fluorescence microscope (EVOS FL Auto, Life Technologies, USA).

### Enzyme-linked immunosorbent assay (ELISA)

Cell culture medium was collected 48 h after EPCs were incubated with 50-μM MPS or 50-μM MPS combined with 1-μM VO-OHpic. The vascular endothelial growth factor (VEGF) concentration was measured with a rat VEGF ELISA kit (Boster, China, EK0540).

### Wound-healing assay

A culture-insert migration assay (ibidi GmbH, Martinsried, Germany) was also used to observe the cell migration of EPCs according to manufacturer’s instructions. First, EPCs were seeded into each chamber of the culture insert and incubated at 37 °C until nearly confluence. Then the insert was removed and cells were incubated in serum-free culture medium supplemented with or without 50-μM MPS or 50-μM MPS combined with 1-μM VO-OHpic. After 12 h and 24 h, the images of the scratch were captured using a microscope (EVOS FL Auto, Life Technologies, USA). Wound healing was analyzed with TScratch software.

### Western blot analysis

EPCs were washed three times with PBS and then covered with 100-μl RIPA lysis buffer (Boster, China, AR0102) containing 1% proteinase inhibitor cocktail for 40 min on ice. Then cell lysis was collected using cell scrapers. The concentration of protein samples was determined by BCA assay kit (Boster, China, AR0146). Then protein samples were separated on 10% of SDS-PAGE (40 μg per lane) and transferred to PVDF membranes (Millipore, Billerica, MA, USA). After blocking with 5% skim milk at room temperature for 1 h, the membranes were incubated overnight with the corresponding primary antibodies at 4 °C. In the second day, the membranes were incubated in 1:5000 diluted horseradish peroxidase-conjugated goat anti-rabbit IgG and goat anti-mouse IgG secondary antibody for 1 h (Boster, China, BA1056). The protein bands were visualized with Western ECL Substrate Kit (Thermo Pierce, USA). Images were captured by Bio-Rad scanner (Hercules, CA), and the density was determined by ImageJ version 1.48.

### Real-time quantitative polymerase chain reaction (RT-qPCR)

Total mRNA expression was extracted with the total RNA extraction kit (Toyobo, Japan) according to the manufacturer’s instructions. Complementary DNA (cDNA) was synthesized from total RNA with first-strand cDNA Synthesis Kit (Toyobo, Japan). Finally, cDNA was amplified using SYBR Green Real-time PCR Master Mix (Toyobo, Japan) with the following cycling conditions: 30 s of polymerase activation at 95 °C, followed by 40 cycles of 95 °C for 5 s and 60 °C for 30 s. The intensity of fluorescent was measured by Bio-Rad Q5 instrument (Bio-Rad Laboratories, CA). GAPDH was selected as the internal control. Sequences of primers used were listed as follows: Nrf2: forward (TAGATGACCATGAGTCGCTT), reverse (CTGTAACTCGGGAATGGAAA); GAPDH: forward (AACATCAAATGGGGTGAGGCC), reverse (GTTGTCATGGATGACCTTGGC).

### Animal experiment

All animal experimental procedures were approved by the Experimental Animal Ethics Committee of Tongji Medical College, Huazhong University of Science and Technology, Wuhan, China. Thirty male Sprague-Dawley rats (7 weeks old, body weight 250–300 g) were used in this study. All animals were housed in hygienic plastic cages at 24 °C in a clean well-ventilated room with 60% humidity and were given free access to conventional rodent chow and water with 12:12 h light and dark cycles. The rats were randomly divided into three groups: control group (*n* = 10), MPS group (*n* = 10), and MPS + VO-OHpic group (*n* = 10). In the MPS group and MPS + VO-OHpic group, rats were intraperitoneally injected with lipopolysaccharide (LPS, 0.2 mg/kg, Sigma-Aldrich, USA) and intramuscularly injected with methylprednisolone (MPS, Pfizer, USA) 40 mg/kg/day for three continuous days. Rats in the control group were injected with the same volume of sterile normal saline intraperitoneally and intramuscularly as control. Rats in the MPS + VO-OHpic group received intraperitoneal injection of VO-OHpic (10 mg/kg) every 2 days. All animals were sacrificed 8 weeks after the first injection.

### Micro-CT analysis

All animals were sacrificed and the femoral heads were collected and evaluated with micro-computed tomography (μCT, viva CT 40, Scanco 274 Medical, Switzerland) at a resolution of 10.5 μm, 100 kV, and 98 μA. Three-dimensional reconstruction and data processing were accomplished using the built-in software. Direct 3D measurement methods were used to calculate the following parameters: bone volume (BV), bone volume per tissue volume (BV/TV), and bone surface/trabecular bone volume (BS/BV).

### Histological and immunohistochemical staining

After CT scanning, femurs were decalcified with 10% EDTA solution for 3 weeks and embedded in paraffin wax. The femoral heads were sectioned at 5-μm thickness in the coronal plane. Some of these sections were stained with hematoxylin and eosin (H&E) to evaluate the trabecular structure, while the others were deparaffinized; antigen retrieved; incubated with anti-CD31, anti-VEGF, and anti-VEGFR2 primary antibodies; and then incubated with the corresponding biotinylated goat anti-rabbit (Boster, China, BA1003) and goat anti-mouse (Boster, China, BA1001) secondary antibodies. Sections were colored with DAB and counterstained with hematoxylin. The images of immunohistochemical staining were analyzed using the software Image-Pro Plus. At least five random fields in the femoral head were selected, and the positive staining was quantified based on the integrated option density (IOD) of target proteins. The corresponding area was also measured, and the mean density was defined as the ratio of integrated optical density to the corresponding area.

### Statistical analysis

All experiments were repeated three times. All the data were represented as mean ± standard deviation (SD). Differences in numerical data between two groups were determined with Student’s two tailed *t* test. One-way ANOVA was used to determine differences among groups more than two followed by a Bonferroni post hoc test. Statistical significance was defined as *p* < 0.05.

## Results

### VO-OHpic significantly attenuates the cytotoxicity of MPS on EPCs

**Fig. 1:**
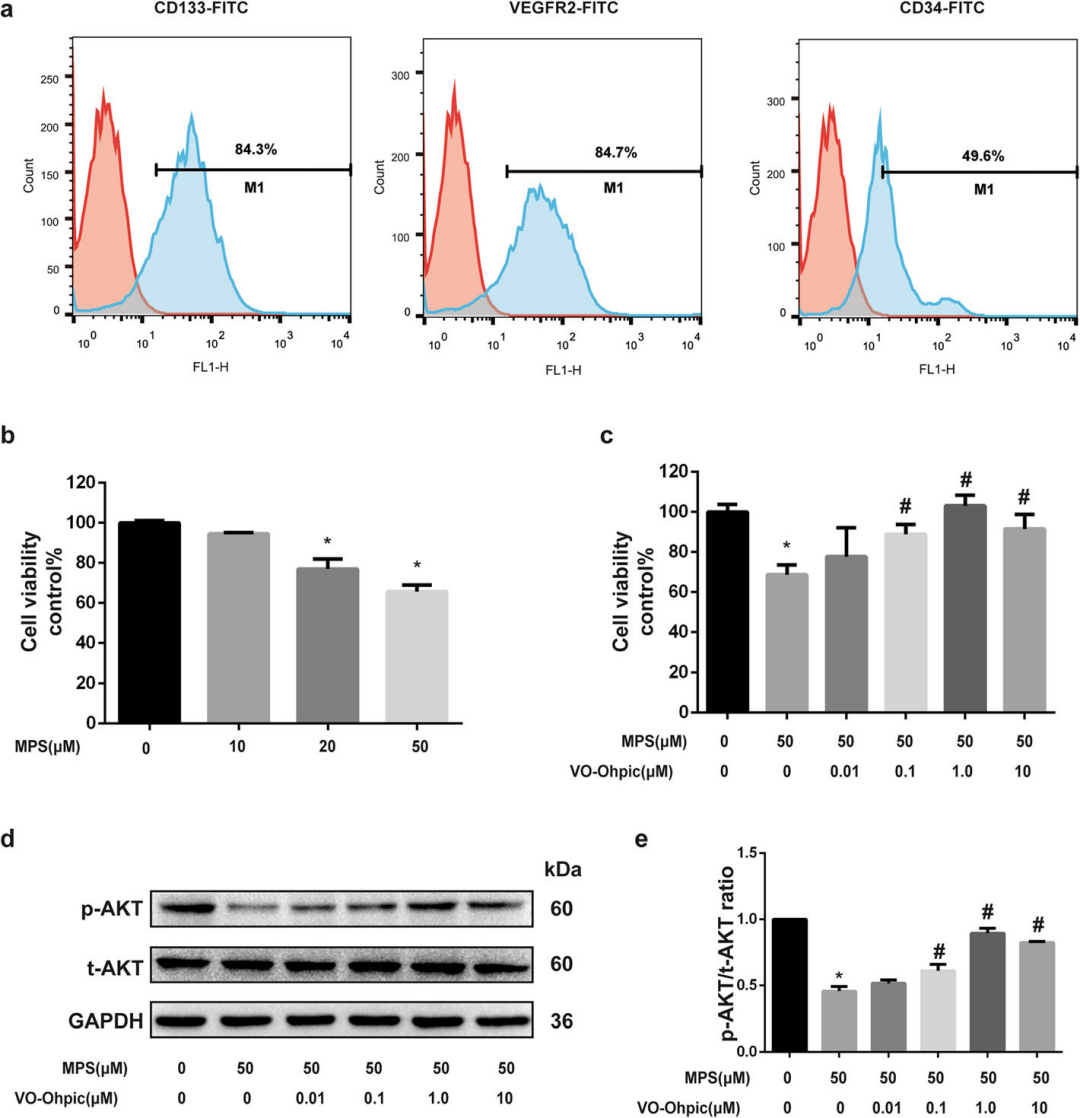
VO-OHpic significantly attenuates the cytotoxicity of MPS on EPCs. **a** The flow cytometry analysis shows that EPCs positively express CD34, VEGFR2, and CD133. **b** The cytotoxicity of MPS on EPCs viability was evaluated with CCK-8 at the concentrations of 0, 10, 20, and 50 μM after 48 h. **c** VO-OHpic effectively attenuates the detrimental effects of MPS (50 μM) on EPCs viability at concentrations of 0.1, 1, and 10 μM. **d** P-AKT and t-AKT protein levels were determined by western blot analysis at 48 h. **e** Band density ratios of p-AKT to t-AKT in the western blots were quantified by densitometry. All experiments were repeated for three times; *P < 0.05 versus control; #*P* < 0.05 versus 50 μM MPS group; Error bar represent SD

The results of cytometry analysis showed that EPCs positively expressed CD34, VEGFR2, and CD133 (Fig. 1a). The results demonstrated that high purity EPCs were obtained and used in the present study [34–36].

To evaluate the cytotoxicity of MPS on EPCs, and determine whether VO-OHpic can attenuate the cytotoxicity of MPS, we perform the CCK-8 assay. The effect of different concentrations of MPS (0, 10, 20, 50 μM) on EPC viability was investigated after 48 h of treatment. The results revealed that MPS dose dependently inhibited the viability of EPCs with the most significant effect at a concentration of 50 μM (Fig. 1b). Then 50-μM MPS was selected in the following studies combined with different concentrations of VO-OHpic (0, 0.01, 0.1, 1, 10 μM). The results showed that VO-OHpic attenuated the cytotoxicity of MPS on EPCs with an optimal concentration of 1 μM (Fig. 1c).

### VO-OHpic significantly abrogates the inhibitory effect of MPS on the AKT signaling pathway in EPCs

Considering that PTEN is the negative regulator of AKT signaling, we investigated the effect of MPS and VO-OHpic on the protein expression and phosphorylation of AKT by western bolt. The results showed that MPS significantly inhibited the phosphorylation of AKT while VO-OHpic attenuated the effect of MPS with an optimal concentration of 1 μM (Fig. 1d, e). Hence, 1-μM VO-OHpic was selected in the following experiments.

### VO-OHpic significantly attenuates MPS-induced apoptosis of EPCs by inhibiting the mitochondrial apoptosis pathway

**Fig. 2:**
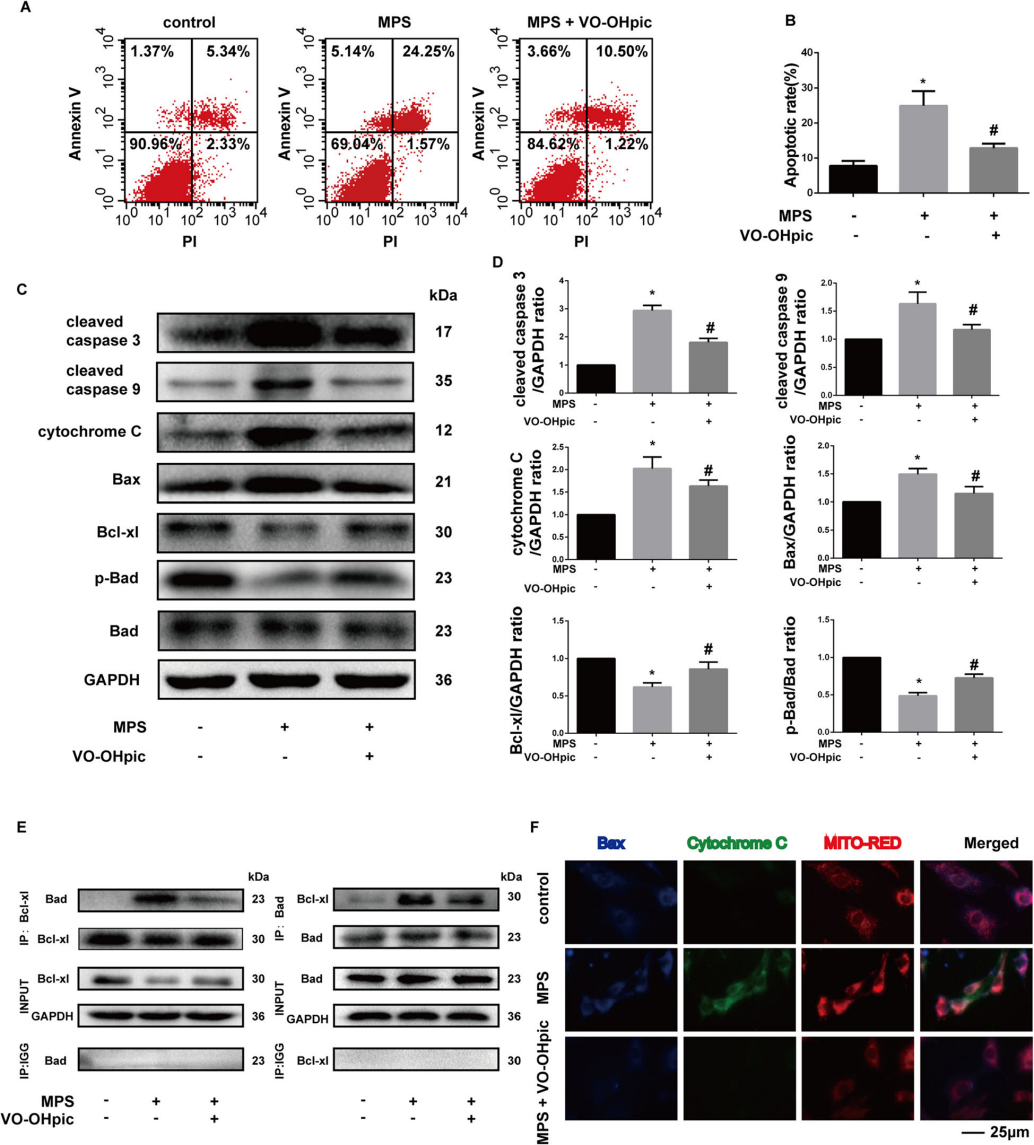
VO-OHpic significantly attenuates MPS-induced apoptosis of EPCs by inhibiting the mitochondrial apoptosis pathway. **a** Flow cytometric analysis of EPCs stained with Annexin V-FITC/PI after treatment with 50-μM MPS with or without 1-μM VO-OHpic for 48 h. **b** Percentage of apoptosis rates were expressed as means ± SD. **c** Protein expressions of cleaved caspase 3, cleaved caspase 9, cytochrome C, Bax, Bcl-xL, p-Bad, and Bad were determined by western blot analysis after treatment with 50-μM MPS with or without 1-μM VO-OHpic for 48 h. **d** Band density ratios of cleaved caspase 3, cleaved caspase 9, cytochrome C, Bax, and Bcl-xL to GAPDH in the western blots were quantified by densitometry. Band density ratios of p-Bad to Bad in the western blots were also quantified by densitometry. **d** The formation of Bad/Bcl-xL pro-apoptotic complex was detected by immunoprecipitation after treatment for 48 h. **f** Both Bax and cytochrome C expression and localization were evaluated by immunofluorescence staining, and the mitochondria were stained by the mito-tracker Red. All experiments were repeated for three times; ∗*P* < 0.05 versus control, #*P* < 0.05 versus 50-μM MPS group. Error bar represents SD

To investigate the roles of mitochondrial apoptosis pathway in the cytotoxicity of MPS and cytoprotective effect of VO-OHpic, we determined the apoptosis rate by flow cytometry and mitochondrial apoptosis-related protein expression by western blot. The apoptosis rate of EPCs was determined with Annexin V-FITC/PI Kit after treatment for 48 h. The average percentage of apoptotic EPCs (early plus late apoptosis) treated with MPS was 24.95 ± 4.21%, which was higher than the control group (7.78 ± 1.39, *P* < 0.05). VO-OHpic attenuated the apoptosis induced by MPS treatment (12.83 ± 1.30, *P* < 0.05) (Fig. 2a, b). Besides, we also found that VO-OHpic could suppress the elevated expression of cleaved caspase-3 and cleaved caspase-9 induced by MPS treatment (Fig. 2c, d). These results indicated that VO-OHpic could attenuate MPS-induced apoptosis. Moreover, we also found that the protein expressions of cytochrome C and Bax, which are key regulators in the mitochondrial apoptosis pathway, were also promoted by MPS treatment, and VO-OHpic treatment significantly reversed the elevated cytochrome C and Bax expressions induced by MPS (Fig. 2c, d). Accordingly, the ratio of p-Bad to Bad was also decreased in the MPS group and the ratio was increased in the MPS + VO-OHpic treatment group which suggested that MPS promoted the activity of the proapoptotic protein Bad while VO-OHpic abrogated the effect (Fig. 2c, d). Moreover, the antiapoptotic protein Bcl-xL expression was inhibited by MPS treatment while VO-OHpic treatment promoted the expression of Bcl-xL (Fig. 2c, d). Heterodimerization of Bad with Bcl-xL is also thought to exert the proapoptotic roles by increasing cytochrome c release from the mitochondria to cytosol [37, 38]. In the present study, we also found that MPS treatment promoted the formation of Bad/Bcl-xL pro-apoptotic complex while VO-OHpic suppressed this process (Fig. 2e). The immunofluorescence results demonstrated that MPS treatment induced the translocation of Bax from cytoplasm to mitochondria and the release of cytochrome C from mitochondria to cytoplasm while VO-OHpic attenuated these changes (Fig. 2f). In summary, VO-OHpic might attenuate MPS-induced apoptosis of EPCs by inhibiting the mitochondrial apoptosis pathway.

### VO-OHpic could restore the normal mitochondrial morphology, suppress excessive ROS generation, and prevent the mitochondrial membrane potential (MMP) collapse induced by MPS

**Fig. 3:**
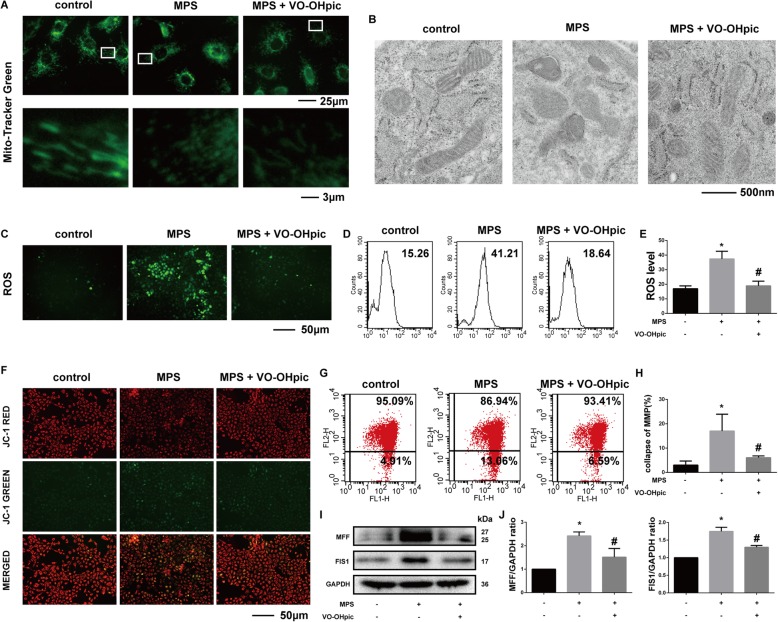
VO-OHpic could restore the normal mitochondrial morphology, suppress excessive ROS generation, and prevent the mitochondrial membrane potential (MMP) collapse induced by MPS. **a** Morphology of the mitochondria was stained with mito-tracker Green, and the representative fluorescence images were captured under a fluorescence microscope after treatment with 50-μM MPS or 50-μM MPS combined with 1-μM VO-OHpic for 48 h. **b** Morphology of the mitochondria was also observed and captured with a TEM after the same treatment. **c** Representative images of EPCs with intracellular ROS stained by the fluorescence probe DCFH-DA after treatment for 48 h. **d** Flow cytometric analysis of ROS production after staining with DCFH-DA. **e** Bar graphs showing the mean fluorescence intensity (MFI) of ROS levels in EPCs. Data are shown as means ± SD. **f** Representative fluorescence images of MMP after incubating with JC-1. Red fluorescence represents JC-1 aggregates in healthy mitochondria whereas green fluorescence represents JC-1 monomer indicating MMP dissipation. Merged images represent co-localization of the JC-1 aggregates and JC-1 monomers. **g** Representative graphs of the flow cytometric analysis after incubating with JC-1. FL1 represents JC-1 green and FL2 represents JC-1 red. **h** MMP were represented as green fluorescence ratio and data were presented as means ± SD. **i** MFF and FIS1 protein levels were determined by western blot analysis after treatment for 48 h. **j** Band density ratios of MFF and FIS1 to GAPDH in the western blots were quantified by densitometry. All experiments were repeated for three times; ∗*P* < 0.05 versus control, #*P* < 0.05 versus 50-μM MPS group. Error bar represents SD

To intuitively observe the pathological changes of mitochondria, we determined the morphology, ROS production, and MMP of mitochondria. With Mito-Tracker Green staining, we found that mitochondria in the EPCs of the control group generally showed a wire-like shape. MPS treatment significantly increased the granulated mitochondria in EPCs, while VO-OHpic restored the normal mitochondrial shape in EPCs (Fig. 3a). Besides, the transmission electron microscopy also revealed that the mitochondrial cristae structure is clear in the control group, but became vague or vanished after MPS treatment, while VO-OHpic partly restored the normal mitochondrial morphology (Fig. 3b). We also found that the control group maintained a relatively low intracellular ROS level (reflected by green fluorescence intensity). However, MPS treatment group showed an increase in ROS generation while VO-OHpic significantly protected EPCs against MPS-induced mitochondrial ROS generation (Fig. 3c). Flow cytometric analysis was also performed to determine the intracellular ROS level quantitatively. The results further supported the conclusion that VO-OHpic could suppress excessive ROS generation induced by MPS (Fig. 3d, e). Collapse in MMP represents cell apoptosis and mitochondrial dysfunction. MPS treatment significantly induced the collapse of MMP (reflected by increased the green fluorescence intensity and decreased the red fluorescence intensity) while VO-OHpic alleviated the detrimental effect of MPS and restored the MMP (Fig. 3f). Flow cytometric analysis was also performed to quantify the percentages of MMP collapse, and the results further proved the effects of MPS and VO-OHpic on MMP in the EPCs (Fig. 3g, h). Several investigations have shown that mitochondrial fission is critical for the mitochondrial homeostasis. Mitochondrial fission factor (MFF) or fission 1 (FIS1)-mediated excessive mitochondrial fission could lead to ROS burst and activation of the mitochondrial apoptosis pathway [39]. In the present study, we also found that MPS promoted MFF and FIS1 protein expressions while VO-OHpic attenuated these changes (Fig. 3i, j). These results revealed that VO-OHpic restored the normal mitochondrial morphology, suppressed the ROS generation, and prevented the MMP collapse of EPCs at least partly by mitigating the excessive mitochondrial fission induced by MPS.

### VO-OHpic exerts protective effects on MPS-regulated angiogenesis of EPCs

**Fig. 4:**
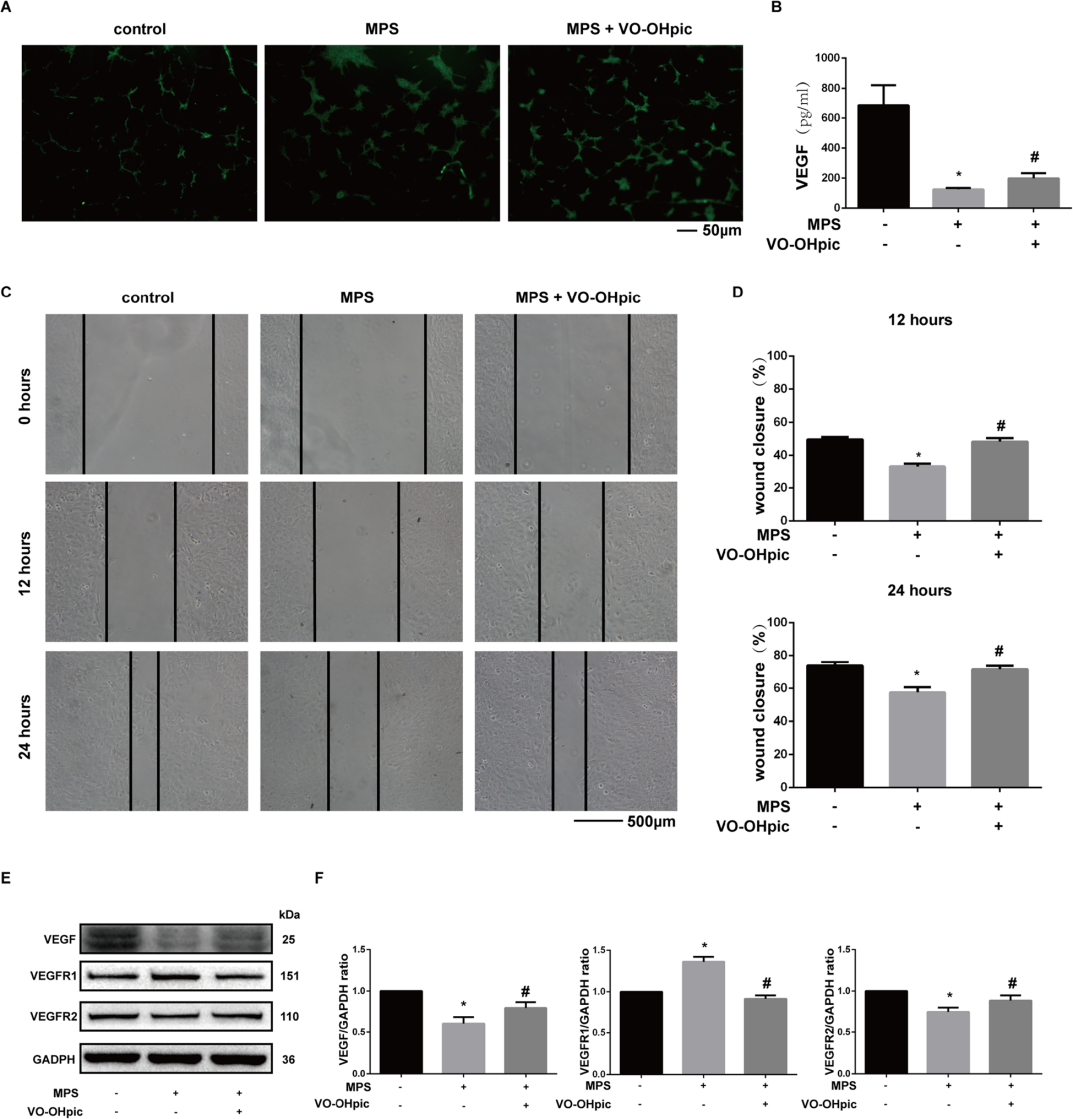
VO-OHpic exerts protective effects on MPS-regulated angiogenesis of EPCs. **a** Representative images of tube formation of EPCs after treatment with 50-μM MPS or 50-μM MPS combined with 1-μM VO-OHpic for 48 h. **b** VEGF protein concentration in the culture medium of EPCs as determined by ELISA after treatment for 48 h. **c** Wound healing assay was performed and representative images were taken after 12 h and 24 h in EPCs treated with 50-μM MPS or 50-μM MPS combined with 1-μM VO-OHpic. **d** The wound closure rate was measured and relatively compared to the control group with no treatment of MPS and VO-OHpic. **e** VEGF, VEGFR1, and VEGFR2 protein levels were determined by western blot analysis after treatment for 48 h. **f** Band density ratios of VEGF, VEGFR1, and VEGFR2 to GAPDH in the western blot analysis were quantified by densitometry. All experiments were repeated for three times; ∗*P* < 0.05 versus control, #*P* < 0.05 versus 50-μM MPS group. Error bar represents SD

To determine the effects of VO-OHpic on angiogenesis of EPCs, we performed Matrigel tube formation assay. The result showed that MPS inhibited the angiogenesis of EPCs while VO-OHpic could protect against the inhibitory effect of MPS (Fig. 4a). The secretion of VEGF, a growth factor that is critical for angiogenesis, was also measured by ELISA. The results revealed that MPS significantly suppressed the secretion of VEGF by EPCs and VO-OHpic increased the secretion of VEGF (Fig. 4b). We also measured the migration ability of EPCs. The results revealed that MPS decreased the migration ability of EPCs while VO-OHpic treatment could partly restore the migration ability of EPCs in both 12 and 24 h (Fig. 4c, d). The protein expression levels of VEGF, VEGFR1, and VEGFR2 were also investigated. The results revealed that the VEGF protein expression was inhibited by MPS treatment, and VO-OHpic treatment promoted the VEGF protein expression (Fig. 4e, f). VEGFR2 is the main receptor during the signal transduction of VEGF while VEGFR1 presents competitive inhibition ability upon VEGF signaling. The results also showed that MPS inhibited VEGFR2 protein expression and promoted VEGFR1 protein expression (Fig. 4e, f).

### VO-OHpic significantly abrogates the inhibitory effect of MPS on the Nrf2 signaling pathway in EPCs

**Fig. 5:**
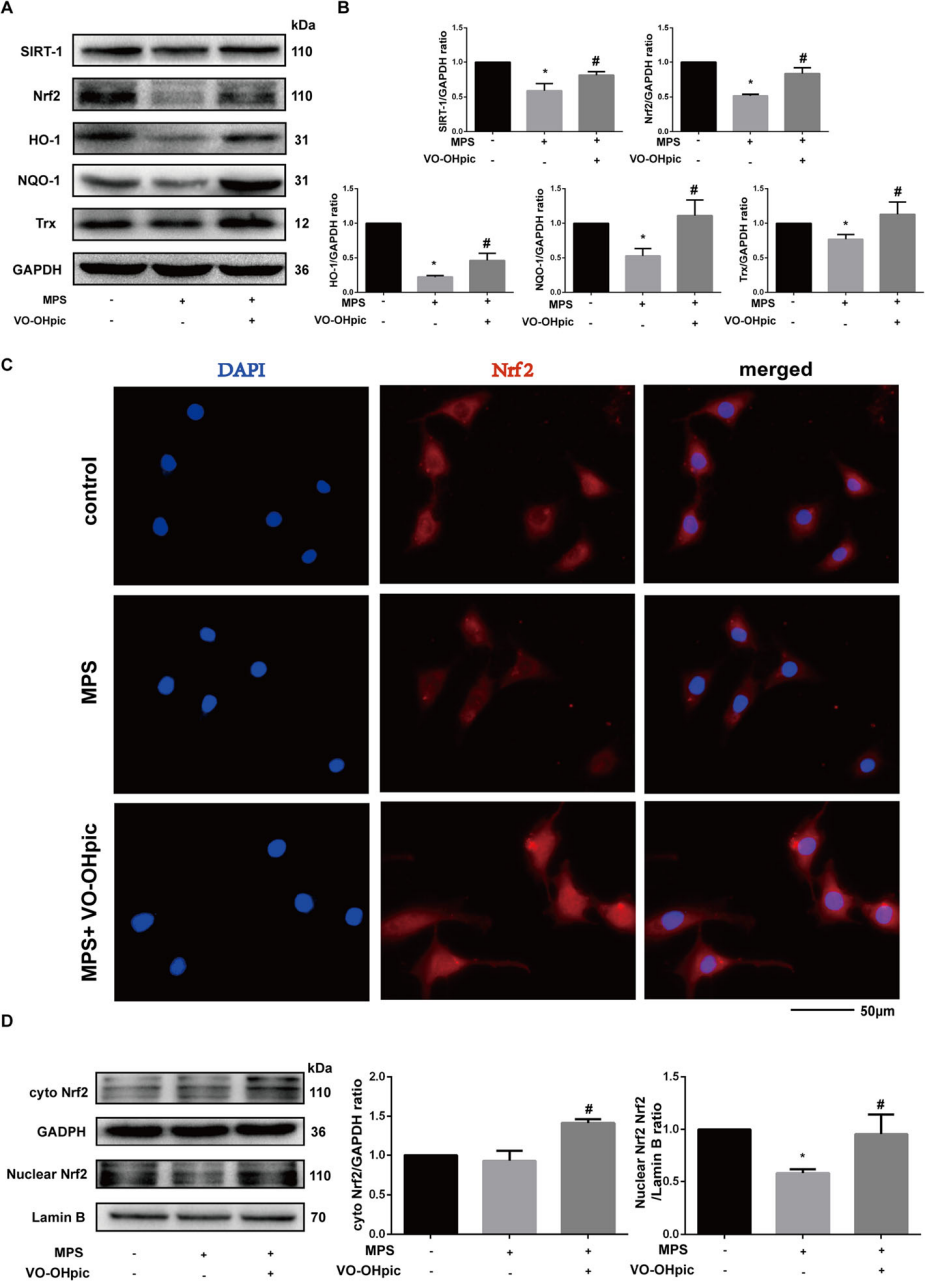
VO-OHpic significantly abrogates the inhibitory effect of MPS on the Nrf2 signaling pathway in EPCs. **a** Sirt1, Nrf2, HO-1, NQO-1, and Trx protein levels in EPCs were determined by western blot analysis after treatment with 50-μM MPS or 50-μM MPS combined with 1-μM VO-OHpic for 48 h. **b** Band density ratios of Sirt1, Nrf2, HO-1, NQO-1, and Trx to GAPDH in the western blots were quantified by densitometry. **c** Immunofluorescence staining of EPCs cultured with 50-μM MPS or 50-μM MPS combined with 1-μM VO-OHpic for 48 h. Nrf2 subcellular localization was determined by immunofluorescence staining for Nrf2 (red) along with DAPI for DNA (blue) at × 400 magnifications. **d** Cytoplasmic Nrf2 protein expression was determined by western blot analysis, and GAPDH was selected as the internal control. Nuclear Nrf2 protein expression was determined by western blot analysis, and Lamin B was selected as the internal control. Band density ratios were also quantified by densitometry. All experiments were repeated for three times; ∗*P* < 0.05 versus control, #*P* < 0.05 versus 50-μM MPS group. Error bar represents SD

Nrf2 signaling is a potent anti-oxidant signaling. In the present study, we investigated the effect of VO-OHpic and MPS on the Nrf2 signaling pathway. The western blot analysis demonstrated that MPS inhibited the protein expression of sirtuin 1 (Sirt1), Nrf2, and its downstream protein HO-1, NQO1, and Trx. VO-OHpic significantly abrogated the effect of MPS and increased the protein expressions of sirt1, Nrf2, HO-1, NQO-1, and Trx (Fig. 5a, b). Besides, the immunofluorescence analysis also testified the results that MPS inhibited Nrf2 protein expression while VO-OHpic promoted both Nrf2 protein expression and the nuclear localization (Fig. 5c). Western blot analysis of both the nuclear and cytoplasmic protein also demonstrated that MPS inhibited the nuclear Nrf2 expression, and VO-OHpic treatment increased both the nuclear and cytoplasmic Nrf2 protein expressions (Fig. 5d).

### Nrf2 activation is required for VO-OHpic-mediated anti-MPS effects in EPCs

**Fig. 6:**
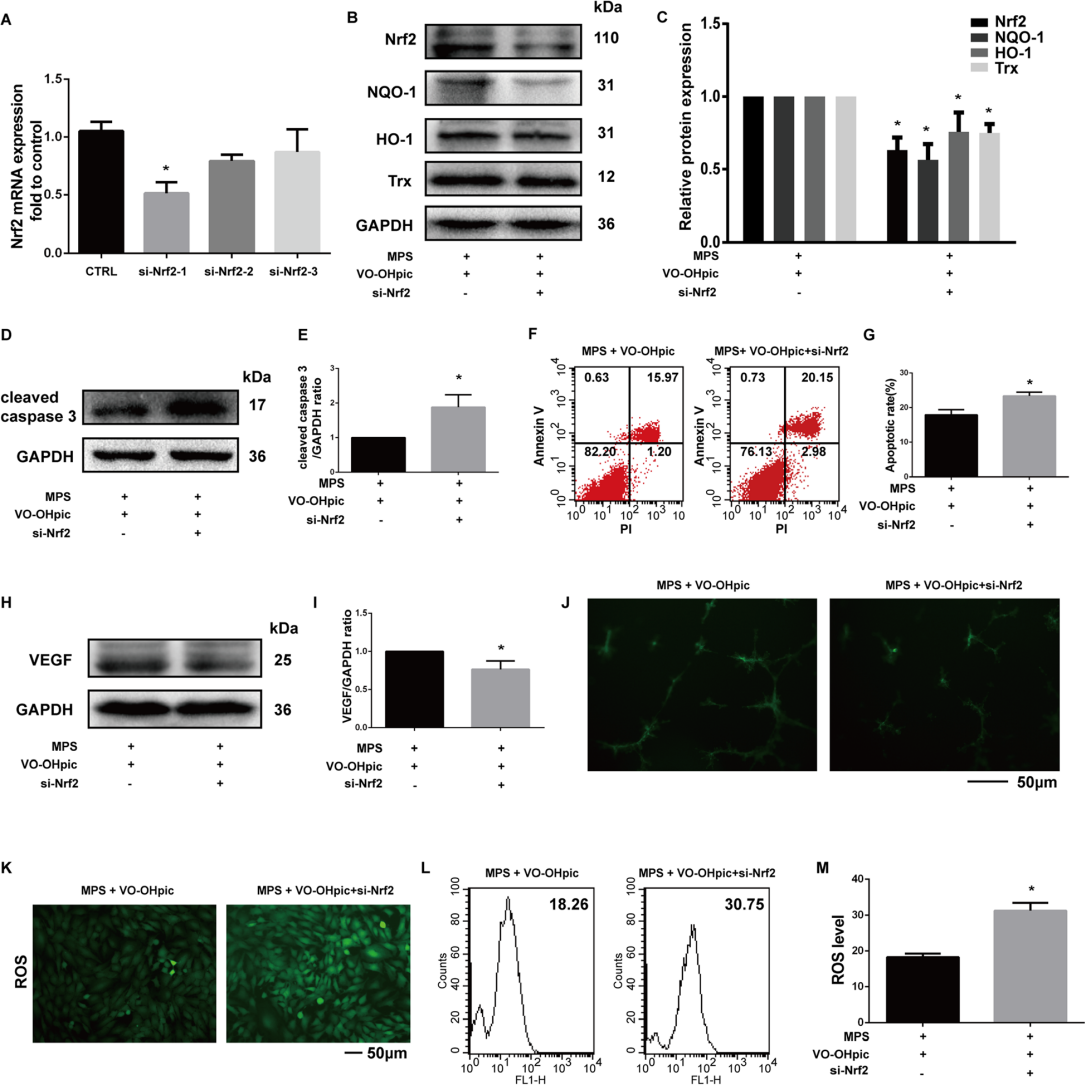
Nrf2 activation is required for VO-OHpic-mediated anti-MPS effects in EPCs. **a** Relative mRNA expressions of Nrf2 as determined by RT-qPCR 2 days after siNrf2 transfection. **b** EPCs transfected with or without siNrf2 for 24 h were then treated with 50-μM MPS or 50-μM MPS combined with 1-μM VO-OHpic for 48 h. Nrf2, NQO-1, HO-1, and Trx protein levels were determined by western blot analysis. **c** Band density ratios of Nrf2, NQO-1, HO-1, and Trx to GAPDH in the western blots were quantified by densitometry. **d** The cleaved caspase 3 protein level was also determined by western blot analysis. **e** Band density ratios of cleaved caspase 3 to GAPDH in the western blots were quantified by densitometry. **f** Flow cytometric analysis of EPCs transfected with or without siNrf2 were stained with Annexin V-FITC/PI after treatment with 50-μM MPS or 50-μM MPS combined with 1-μM VO-OHpic for 48 h. **g** Percentage of apoptosis rates was expressed as means ± SD. **h** The VEGF protein level was determined by western blot analysis. **i** Band density ratios of VEGF to GAPDH in the western blots were quantified by densitometry. **j** Representative images of tube formation of EPCs in 12 h. EPCs were pre-transfected with and without siNrf2 for 24 h and then treated with 50-μM MPS combined with 1-μM VO-OHpic before seeded into Matrigel. **k** Representative images of EPCs transfected with or without siNrf2 with intracellular ROS stained by the fluorescence probe DCFH-DA after treatment for 48 h. **l** Flow cytometric analysis of ROS production after staining with DCFH-DA. **m** Bar graphs showing the mean fluorescence intensity (MFI) of ROS levels in EPCs. Data are shown as means ± SD. All experiments were repeated for three times; ∗*P* < 0.05 versus EPCs without siNrf2 transfection. Error bar represents SD

The previous results have shown that VO-OHpic activated the Nrf2 signaling pathway, inhibited the apoptosis and ROS generation of EPCs induced by MPS, and rescued the angiogenic differentiation of EPCs suppressed by MPS. To further elucidate whether VO-OHpic exerted these effects via Nrf2, we used siRNA to knockdown Nrf2 expression in EPCs. The RT-qPCR results showed that si-Nrf2-1 significantly suppressed Nrf2 mRNA expression, so si-Nrf2-1 were used in following experiments (Fig. 6a). The western blot results showed that si-Nrf2 potently suppressed the protein expression level of Nrf2 and its downstream proteins including NQO-1, HO-1, and Trx (Fig. 6b, c). The results also showed that si-Nrf2 significantly increased the cleaved caspase 3 protein expression (Fig. 6d, e) and EPC apoptosis (Fig. 6f, g). Besides, we also found that si-Nrf2 transfection potently inhibited the VEGF protein expression and angiogenesis of EPCs (Fig. 6h, j). In addition, the immunofluorescence and flow cytometry analysis also showed that si-Nrf2 significantly increased the ROS in EPCs (Fig. 6k, m).

### VO-OHpic alleviates MPS-induced osteonecrosis of the femoral head in rats

**Fig. 7:**
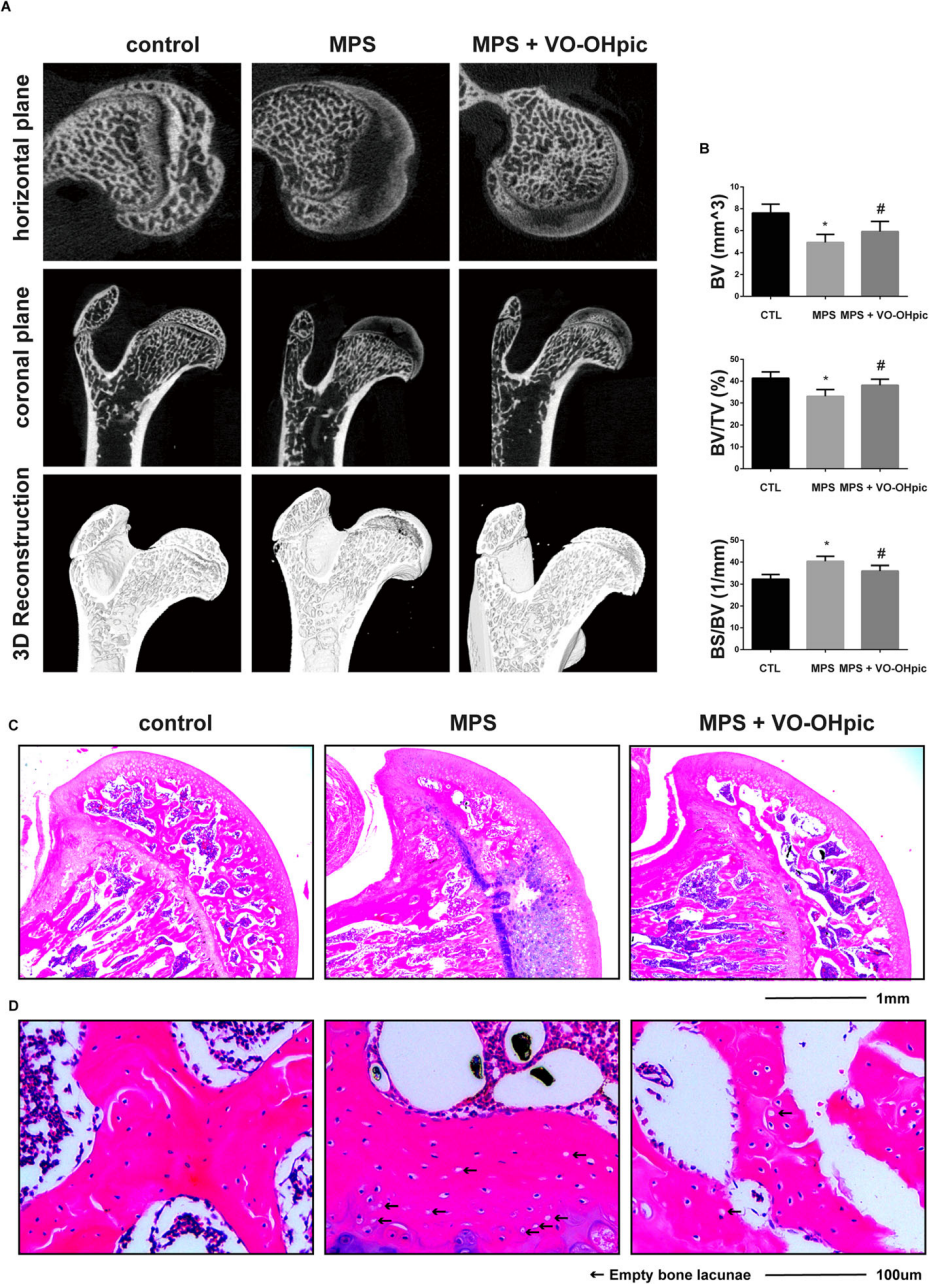
VO-OHpic alleviates MPS-induced osteonecrosis of the femoral head in rats. **a** Representative micro-CT images of the femoral heads (horizontal plane, coronal plane, and 3D reconstruction images). **b** The microstructural parameters of subchondral trabecular bone including bone volume (BV), bone volume/total volume (BV/TV), and bone surface/trabecular bone volume (BS/BV) were quantitatively evaluated in rats treated with MPS or treated with MPS and VO-OHpic. ∗*P* < 0.05 versus control, #*P* < 0.05 versus MPS group. Error bar represents SD. **c** HE staining of the rat femoral head was shown in the coronal plane (scale bar = 1 mm). **d** Local magnification showed that more empty bone lacunae were present in the MPS group compared to the control, while in the MP + VO-OHpic group, there is less empty bone lacunae compared to the MPS group (scale bar = 100 μm). Each group consisted of 10 rats/20 femoral heads (*N* = 20)

Micro-CT and HE staining were used to evaluate the progression of ONFH. Micro-CT showed that MPS significantly induced ONFH as evidenced by the bone resorption in the femoral head. The micro-CT also showed that MPS decreased the BV, BV/TV but increased the BS/BV of the femoral head (Fig. 7a, b). Treatment with MPS plus VO-OHpic significantly abrogated the deleterious effects of MPS on the bones, resulted in increased BV and BV/TV, decreased BS/BV, and reduced bone resorption in the femoral head (Fig. 7a, b). The HE staining also showed that MPS significantly increased the number of empty bone lacuna, while fewer empty bone lacuna was detected in the rats treated with MPS and VO-OHpic (Fig. 7c, d).

### VO-OHpic significantly promotes angiogenesis in MPS-induced osteonecrosis of the femoral head

**Fig. 8:**
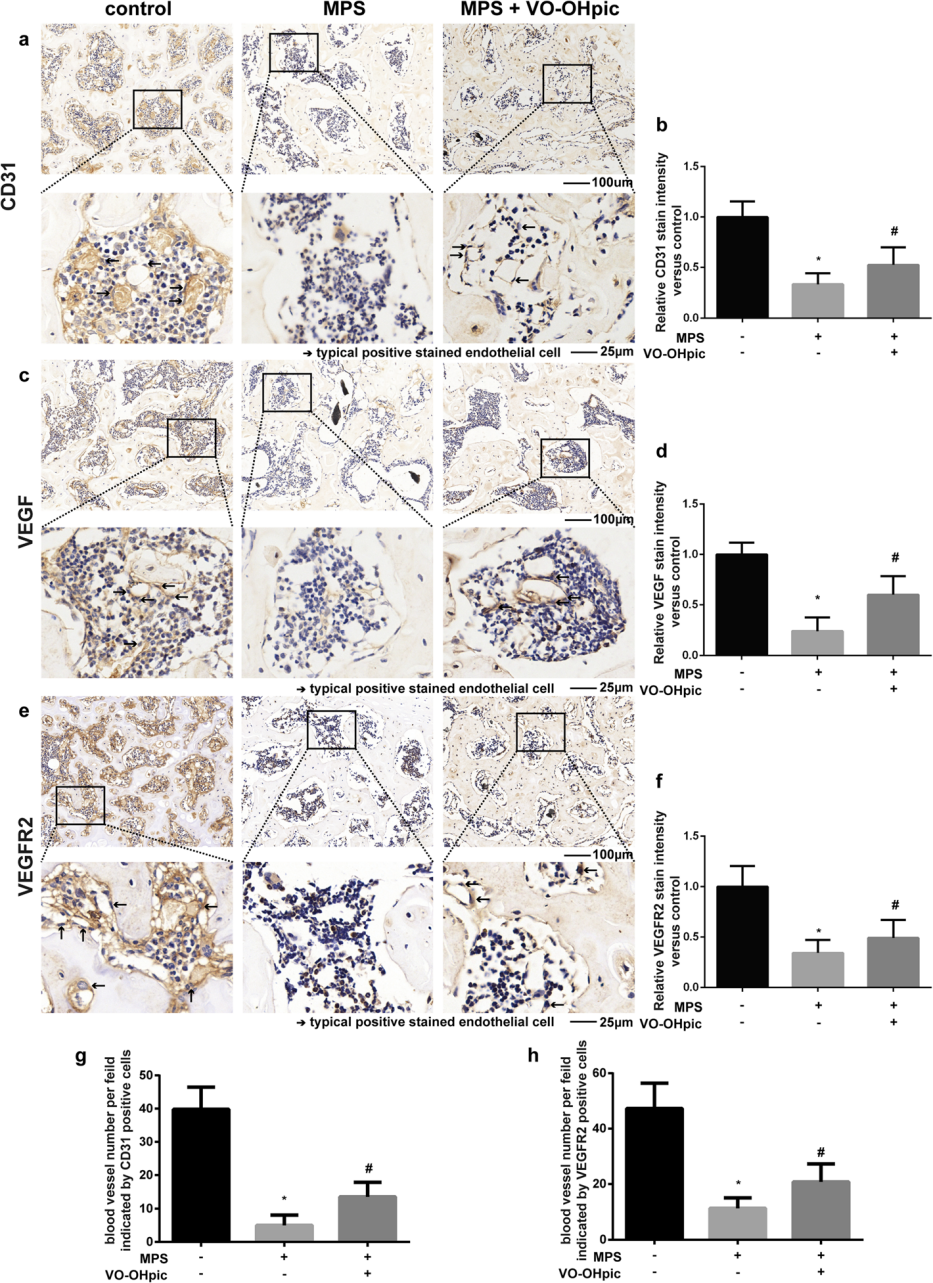
Immunohistochemical staining of the sections of representative rat femoral heads. **a** Representative images of CD31 staining in the control, MPS, and MPS + VO-OHpic groups. **b** Relative CD31 expression was measured with the ImageJ software. ∗*P* < 0.05 versus control, #*P* < 0.05 versus MPS group. Error bar represents SD. **c** Representative images of VEGF staining in the control, MPS, and MPS + VO-OHpic groups. **d** Relative VEGF expression was measured with the ImageJ software. ∗*P* < 0.05 versus control, #*P* < 0.05 versus MPS group. Error bar represents SD. **e** Representative images of VEGFR2 staining in the control, MPS, and MPS + VO-OHpic groups. **f** Relative VEGFR2 expression was measured with the ImageJ software. **g** CD31-positive blood vessel number per field under × 200 magnification. **h** VEGFR2-positive blood vessel number per field under × 200 magnification. ∗*P* < 0.05 versus control, #*P* < 0.05 versus MPS group. Error bar represents SD. Each group consisted of 10 rats/20 femoral heads (*N* = 20)

To investigate the effect of MPS and VO-OHpic on the blood supply of the femoral head, immunohistochemical staining of CD31, VEGF, and VEGFR2 were performed to evaluate the function of endothelial cells and localized the blood vessels. The immunohistochemical staining revealed that MPS decreased CD31, VEGF, and VEGFR2 expressions, indicating the inhibitory effects of MPS on the function of endothelial cells and the blood supply in the rat femoral heads (Fig. 8a, f). Treatment with MPS and VO-OHpic significantly promoted CD31, VEGF, and VEGFR2 protein expressions in the subchondral bone trabeculae of rat femoral head compared with MPS-treated group (Fig. 8a, f). Also, we evaluated the blood vessel density by counting the number of blood vessels (circles that formed by CD31- and VEGFR2-positive cells) per field in each group under × 200 magnification. The results showed that the blood vessels’ density is decreased in ONFH group while VO-OHpic can partly restored the blood supply (Fig. 8g, h).

## Discussion

The adverse effects of hypercortisolism on bone were first realized as early as 1932. More than 50% of GC users develop significant bone loss, and about 40% of GC users develop different degrees of osteonecrosis [40, 41]. GC administration has already been shown to be the most common cause of nontraumatic ONFH [42]. Many different pathophysiological mechanisms for GC-associated ONFH have been proposed. However, the pathogenesis of GC-associated ONFH is still unclear. The most compelling hypothesis for GC-associated ONFH is vascular hypothesis which presumes that damaged vessels and decreased local blood flow in the femoral head play a pivotal role in the pathogenesis of ONFH [3, 4]. The EPCs play an important role in maintaining the normal structure and function of the blood vessels [33]. Interestingly, autologous implantation of EPCs could significantly promote the neovascularization and bone regeneration in GC-associated ONFH in rabbits [43]. Moreover, the number and function of EPCs were inhibited in GC-associated ONFH patients [6, 7]. Thus, EPCs might be a promising target for the prevention and therapy of ONFH.

Vanadium compounds have been recognized as broad spectrum inhibitors of phosphatase enzymes. And selective inhibition of certain member of the protein tyrosine phosphatase family can be achieved by oxidation of the active site cysteine thiol. VO-OHpic is a vanadyl compound in complex to hydroxypicolinic acid that specifically inhibits PTEN’s enzymatic activity in vitro and in vivo. VO-OHpic has many advantages compared with other inhibitors. Firstly, VO-OHpic is highly selective to PTEN. Secondly, VO-OHpic inhibits PTEN with low nanomolar concentrations. Lastly, the inhibition of PTEN by VO-OHpic is reversible [16]. Thus, VO-OHpic seems to be a very attractive therapeutic option.

The extrinsic death receptor pathway and the intrinsic mitochondrial pathway are two major systems that could activate the expression of caspases and subsequent cell apoptosis [44]. Previous studies have reported the pro-apoptosis effects of excess GC on osteoblasts [45], osteocytes [46], and endothelial cells [47]. In the present study, we firstly reported that excess GC could induce EPC apoptosis through activating mitochondrial pathway. We proved that inhibition of PTEN by VO-OHpic is able to inactivate the caspase system and protect EPCs from MPS-induced apoptosis through suppressing the mitochondrial apoptosis pathway.

We also found that the mitochondrial morphology, ROS production, and MMP were significantly abnormal when treated with MPS. Those results are consistent with previous studies that report excess GC would induce superoxide production and dysfunction of vascular endothelial cells [48]. As far as we know, we are the first to study the elucidation of the MPS-induced mitochondrial dysfunction of EPCs. VO-OHpic was able to maintain a normal mitochondrial structure, decrease the mitochondrial ROS generation, and restore a normal MMP. We also detected excessive mitochondrial fission in MPS-treated EPCs while VO-OHpic attenuated these effects. According to previous studies [49, 50], the excess mitochondrial fission might be responsible for the abnormal mitochondrial morphology and ROS burst.

PTEN is considered as a negative regulator of angiogenesis in many kinds of tumors [51, 52]. Our in vitro studies also showed that MPS inhibited the angiogenesis and migration of EPCs while VO-OHpic could protect against the inhibitory effects of MPS and promoted the angiogenesis and migration of EPCs. In vivo experiment, intraperitoneal injection of VO-OHpic preserved the local blood supply of femoral head. Together, our study demonstrated the angiogenic effect of VO-OHpic.

We found Nrf2, a transcription factor that controls the expression of many antioxidant enzymes [53], is critical for the protective effect of VO-OHpic. The importance of Nrf2 further elucidated the important roles of oxidative stress in the MPS-induced EPC dysfunction. However, the influence of Nrf2 on angiogenesis is still controversial. Nrf2 positively or negatively regulate the angiogenesis under different situation [54, 55]. In our study, we demonstrated that Nrf2 positively regulated the angiogenesis in EPCs and ONFH.

Finally, we investigated the effect of VO-OHpic on MPS-induced ONFH in vivo. Micro-CT revealed that MPS significantly induced ONFH in rats while VO-OHpic abrogated the deleterious effects of MPS on the bones and attenuated the ONFH in rats. HE staining also showed that VO-OHpic significantly reduced the number of empty bone lacuna induced by MPS in the femoral head. Those results confirmed that inhibition of PTEN by VO-OHpic might be a promising strategy for the prevention and therapy of ONFH.

As far as we know, the present study was the first to propose the prevention and treatment of GC-associated ONFH by inhibiting PTEN and also the first to investigate the effect of PTEN inhibitor VO-OHpic in GC-associated ONFH. We were able to show that MPS promoted the mitochondrial apoptosis and ROS generation of EPCs, induced collapse of the MMP, and inhibited the angiogenesis and migration of EPCs. VO-OHpic was able to reverse the harmful effects of MPS by activating the Nrf2 signaling pathway and inhibiting the mitochondrial apoptosis pathway. Our in vivo studies also revealed that VO-OHpic was able to attenuate MPS-associated ONFH in rats.

## Conclusion

In conclusion, we proved that inhibition of PTEN by VO-OHpic might be a promising strategy in the prevention and treatment of associated ONFH.

## Data Availability

All data of the current study are available from the corresponding author on reasonable request.

## Abbreviations

GC: Glucocorticoid
ONFH: Osteonecrosis of the femoral head
EPCs: Endothelial progenitor cells
PTEN: Phosphatase and tensin homolog
MPS: Methylprednisolone
Nrf2: NF-E2-related factor 2
PI3K: Phosphatidylinositol 3-kinase
ARE: Antioxidant response element
HO-1: Heme oxygenase 1
NQO-1: Quinone dehydrogenase 1
Trx: Thioredoxin
ROS: Reactive oxygen species
MMP: Mitochondrial membrane potential
VEGF: Vascular endothelial growth factor
